# Predicting Psychological Symptoms When Facebook’s Digital Well-being Features Are Used: Cross-sectional Survey Study

**DOI:** 10.2196/39387

**Published:** 2022-08-29

**Authors:** Tamara Barsova, Zi Gi Cheong, Ann R Mak, Jean CJ Liu

**Affiliations:** 1 Yale-NUS College Singapore Singapore; 2 Centre for Sleep and Cognition NUS Yong Loo Lin School of Medicine Singapore Singapore; 3 Centre for Trusted Internet and Community National University of Singapore Singapore Singapore; 4 Lee Kuan Yew School of Public Policy National University of Singapore Singapore Singapore

**Keywords:** mental health, social media, digital well-being, depression, anxiety, stress

## Abstract

**Background:**

Prior research has linked social media usage to poorer mental health. To address these concerns, social media platforms have introduced digital well-being tools to help users monitor their engagement. Nonetheless, little is known about the effectiveness of these tools.

**Objective:**

In this study, we focused on Facebook to assess users’ awareness and usage of the following six Facebook well-being tools: the *Unfollow*, *Snooze*, *Off-Facebook Activity*, *Your Time on Facebook*, *Set Daily Reminders*, and *Notification Settings* features. Additionally, we examined whether the use of these tools was associated with better mental health outcomes.

**Methods:**

We conducted a cross-sectional survey of 598 Facebook users. The survey comprised questions about (1) baseline Facebook use, (2) the adoption of Facebook’s digital well-being tools, and (3) participant demographics. These were used to predict the primary outcome measure—scores on the 21-item Depression, Anxiety, and Stress Scale.

**Results:**

Most participants (580/598, 97%) knew about Facebook’s digital well-being tools, but each tool was used by only 17.4% (104/598) to 55.5% (332/598) of participants. In turn, the use of two tools was associated with better well-being; although participants who spent more time on Facebook reported higher levels of depression, anxiety, and stress, those who managed their feed content or notifications by using the *Unfollow* or *Notification Settings* features had lower scores on each of these measures. However, the use of the *Snooze*, *Off-Facebook Activity*, *Your Time on Facebook*, or *Set Time Reminder* features was not associated with lower depression, anxiety, or stress scores.

**Conclusions:**

Of the 6 Facebook digital well-being tools, only 2 were associated with better mental health among users. This underscores the complexity of designing social media platforms to promote user welfare. Consequently, we urge further research into understanding the efficacy of various digital well-being tools.

**Trial Registration:**

ClinicalTrials.gov NCT04967846; https://clinicaltrials.gov/ct2/show/study/NCT04967846

## Introduction

### Background

Over the past decade, social media platforms have been scrutinized for their potential impact on mental health. Among the general public, claims about social media harms have been widely publicized in both television documentaries [1] and whistleblower accounts [2]. Within the academic literature, multiple studies have also linked social media usage to symptoms of depression [3,4], psychological distress [5], poorer well-being [5,6], and lower self-esteem [7].

Two theories have been proposed to explain why social media platforms may compromise mental health [1,2]. First, such platforms allow users to compare themselves with celebrities or peers whose web-based posts portray more ideal lives than those of typical users [8,9]. This form of upward social comparison may result in users feeling worse about themselves, placing them at risk for poorer mental health [8,9]. Second, social media platforms are designed to draw users’ attention for as long as possible [10,11]. In turn, this allure may result in excessive social media consumption, again impairing well-being [10,11].

To address public concerns about these social media harms, app developers have introduced digital well-being features to help users manage their engagement [12,13]. Nonetheless, it remains unclear (1) whether users know or use these features and (2) whether the use of these features predicts better psychological well-being. Consequently, this study examines these questions by focusing on Facebook as a case study.

### Facebook’s Digital Well-being Features

With 2.9 billion users worldwide, Facebook is the most widely used social networking platform in the world [14]. Given its popularity, it has also been the focus of most research studies that document the link between social media usage and poorer mental health [7,9,15]. As a result, Facebook developers consulted mental health experts and launched a series of digital well-being features, with the high-level goal of making subsequent Facebook usage “intentional, positive and inspiring” [16].

Facebook’s digital well-being features broadly address the two proposed theories for social media harms. First, to minimize the amount of social comparisons, several features allow users to curate the content that they see. For example, the *Unfollow* option allows users to hide posts from selected friends, pages, or groups, while the *Snooze* option hides these posts for a 30-day duration [17]. Further, the *Off-Facebook Activity* feature allows users to customize how the platform integrates information from external apps to customize their feeds [17].

Based on prior surveys, content curation features seem to be adopted when users want to avoid friends’ boastings, inappropriate posts (eg, racist content), content that they disagree with (eg, on account of political ideology), or excessive and irrelevant posts [18-21]. In turn, deploying these features can cause users to feel unburdened [22]. Consequently, we sought to examine whether the adoption of these features predicts better mental health.

In the second category, a separate set of digital well-being features enables users to monitor their usage patterns and curb excessive use. For example, the *Your Time on Facebook* feature displays the amount of time that a user has spent on Facebook over the past week, while the *Set Daily Reminders* feature notifies users when a predetermined cutoff has been reached (eg, 45 minutes of Facebook use) [17]. Additionally, the *Notification Settings* feature allows users to manage the in-app notifications that they receive, minimizing the amount of content that draws the users’ attention.

As we are not aware of any study linking Facebook’s digital well-being tools to mental health, we conducted a cross-sectional survey to address our two primary aims. First, we sought to document the extent to which Facebook users know and use the six outlined features—the (1) *Unfollow*, (2) *Snooze*, (3) *Off-Facebook Activity*, (4) *Your Time on Facebook*, (5) *Set Daily Reminders*, and (6) *Notification Settings* features. Second, we sought to replicate previous findings that linked Facebook usage with poorer mental health and examine whether participants’ use of the well-being features was associated with better outcomes.

## Methods

### Study Design and Population

The participants were 608 Facebook users who were recruited from Amazon’s web-based panel (Mechanical Turk) in June 2021. All participants met the following eligibility criteria: (1) individuals aged 21 years or older, (2) individuals who were proficient in English, (3) individuals based in the United States, and (4) individuals with a positive track record on the platform (human intelligence task approval rate: >95%; number approved: >500).

### Ethics Approval

Participants gave their written consent in accordance with the Declaration of Helsinki, and were given a nominal sum of US $0.50 upon study completion. This study was approved by the Yale-NUS College Ethics Review Committee (approval number: 2021-CERC-001) and was preregistered on ClinicalTrials.gov (trial number: NCT04967846).

### Predictor Variables

Predictor and outcome variables were measured through a 10-minute survey that was hosted on the Qualtrics website (Qualtrics International Inc) [23]. The questions were written for a seventh-grade reading level and were pilot-tested before this study.

#### Baseline Facebook Usage

The first set of questions captured participants’ baseline Facebook usage. Following studies that linked Facebook use to mental health, participants estimated the daily number of hours that they spent on Facebook over the past week [24].

To provide a context for these metrics, participants also reported how frequently they engaged in the following nine Facebook activities: reading their news feed, posting status updates, posting photos, posting original content, browsing friends’ timelines, viewing friends’ photos, commenting on friends’ posts, sharing friends’ content, and using Facebook Messenger [24]. These were rated by using 7-point scales anchored with “never” and “more than once a day.”

#### Awareness and Adoption of Facebook Well-being Features

Central to this study, participants also reported their awareness and adoption of the following six Facebook digital well-being tools: the *Unfollow*, *Snooze*, *Off-Facebook Activity*, *Your Time on Facebook*, *Set Daily Reminders*, and *Notification Settings* features.

First, participants were shown screenshots of each feature and reported whether they had heard of the features (“yes” or “no”). If participants responded “yes,” they were then asked if they had used the features (“yes” or “no”). For features that were designed for repeated use (*Unfollow*, *Snooze*, *Off-Facebook Activity*, and *Your Time on Facebook*), participants reported how frequently they used each feature (using a 5-point scale anchored with “never” and “daily”).

#### Demographics

As the final category of predictors, participants reported their age, gender, race, religion, marital status, education level, employment status, family income, household size, and living setting.

### Outcome Measures

As an assay of mental health, participants completed the 21-item Depression, Anxiety, and Stress Scale (DASS-21) [25]. The DASS-21 has been well validated and widely used, consisting of 7 items for each of the following subscales: depression (eg, “I couldn’t seem to experience any positive feelings at all” and “I found it difficult to work up the initiative to do things”; Cronbach α=.87), anxiety (eg, “I was aware of dryness of my mouth” and “I felt I was close to panic”; Cronbach α=.89), and stress (eg, “I found it hard to wind down” and “I tended to over-react to situations”; Cronbach α=.89). Each item was rated on a 4-point scale (ranging from “0: did not apply to me at all” to “3: applied to me very much or most of the time”), and scores were summed and multiplied by 2.

### Statistical Analysis

As part of data cleaning, we first verified that participants had read the questions through two verification items that asked participants to check boxes as instructed (modeled after the widely used CAPTCHA technique on the internet) [26]. Of the 608 participants, 10 (1.6%) failed the verification and were removed from the data set, resulting in a final sample of 598 participants. We then summarized participants’ baseline characteristics by using medians (with IQRs) and counts (with percentages). For count data, error margins for the 95% CIs of proportions were computed by using the *prop.test* function in R (R Foundation for Statistical Computing).

As the primary analyses, we ran a series of linear regression models, using each DASS-21 subscale score (depression, anxiety, and stress) as an outcome measure. In the first model, we sought to replicate the oft-reported link between one’s duration of Facebook use and poorer mental health [8]. To this end, we entered the number of hours that participants spent using Facebook as a predictor. As the visual inspection of the data revealed a right-skewed distribution, this variable was log-transformed to achieve linearity (model 1).

In the second model, we addressed this study’s primary aim—examining whether the adoption of Facebook’s well-being features predicted better mental health (having controlled for the duration of Facebook use). Correspondingly, model 1 was repeated with 6 additional predictors that coded for the use of each feature (*Unfollow*, *Snooze*, *Off-Facebook Activity*, *Your Time on Facebook*, *Set Daily Reminders*, and *Notification Settings*; model 2). For each predictor, nonusage was coded as “0” and usage was coded as “1.”

Finally, we assessed the robustness of our findings by repeating model 2 with the inclusion of demographic variables (age, gender, race, religion, marital status, education, employment, family income, household size, and living setting; model 3).

Across the models, the type 1 decision-wise error rate was controlled at an level of .05, with adequate statistical power (0.80) for detecting small effect sizes (*f*^2^=0.05). All statistical analyses were carried out on SPSS 27 (IBM Corporation) and R version 4.0.3 (R Foundation for Statistical Computing).

## Results

### Participant Characteristics and Baseline Facebook Usage

**Figure 1:**
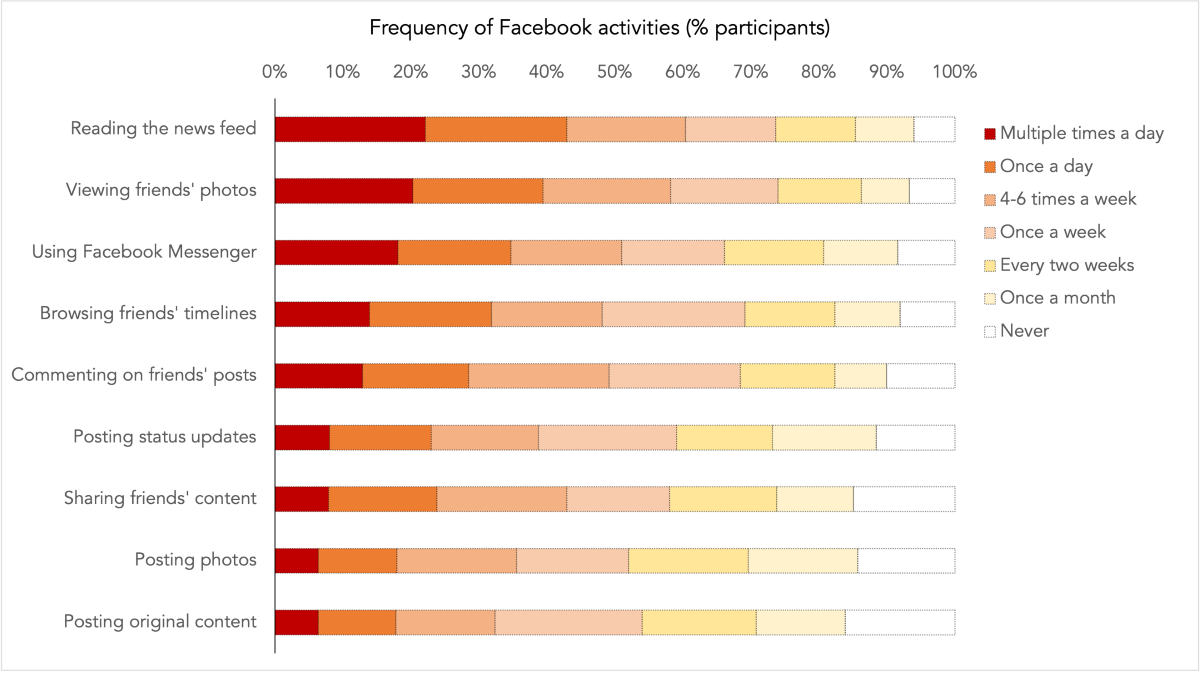
For each of the nine Facebook activities, participants rated whether they used it (1) multiple times a day, (2) once a day, (3) 4 to 6 times a week, (4) once a week, (5) every 2 weeks, (6) once a month, or (7) never. Each horizontal bar indicates the percentage of participants who chose each option.

Of the 598 participants, 309 (51.6%) were aged <35 years, and slightly over half of the participants (360/598, 60.2%) self-identified as men (Table 1). In terms of baseline Facebook usage, participants reported using the platform for a median of 3 (IQR 1-7) hours each day in the preceding week. Further, 289 (48.3%, 95% CI 44.3%-52.3%) participants accessed Facebook multiple times a day, while 130 (21.7%, 95% CI 18.4%-25%) logged in once a day (Table 1). On Facebook, participants were most likely to view a friend’s photos or to read the news feed (Figure 1).

**Table 1:** Baseline characteristics of survey respondents (N=598).

Characteristic				Participants, n (%)
**Age group (years)**				
	<35			309 (51.6)
	≥35			289 (48.4)
**Gender**				
	Women			237 (39.6)
	Men			360 (60.2)
	Nonbinary/third gender			1 (0.2)
**Race**				
	White			475 (79.4)
	Black or African American			84 (14)
	**Other**			39 (6.5)
		Asian	20 (3.3)	
		American Indian or Alaska Native	9 (1.5)	
		2 or more races	6 (1)	
		Other	4 (0.7)	
**Religion**				
	No religion			81 (13.5)
	Christianity (Protestant)			370 (61.9)
	Christianity (Catholic)			107 (17.9)
	**Other**			121 (20.2)
		Buddhism	17 (2.8)	
		Hinduism	10 (1.7)	
		Islam	3 (0.5)	
		Other	10 (1.7)	
**Marital status**				
	Married/partnered			464 (77.6)
	Single			116 (19.4)
	**Other**			18 (3)
		Divorce	16 (2.7)	
		Separated	2 (0.3)	
**Education level**				
	Less than high school			1 (0.2)
	High school diploma or equivalent			26 (4.3)
	Associate degree			26 (4.3)
	Bachelor’s degree			398 (66.6)
	Some college but no degree			37 (6.2)
	Postgraduate degree (eg, master’s degree or doctoral degree)			68 (11.4)
	Professional degree (eg, JD or MD)			42 (7)
**Employment status**				
	Full-time: 40 hours or more per week			507 (84.8)
	**Not full-time**			91 (15.1)
		Part-time: up to 39 hours per week	42 (7)	
		Self-employed	25 (4.2)	
		Retired	8 (1.3)	
		Unemployed; looking for work	7 (1.2)	
		Unemployed; not looking for work	5 (0.8)	
		Unable to work	2 (0.3)	
		Student	2 (0.3)	
**Family income level (US $)**				
	<30,000			68 (11.4)
	30,000-49,999			149 (24.9)
	50,000-74,999			212 (35.5)
	75,000-99,999			115 (19.2)
	≥100,000			54 (9)
**Household size (number of household members)**				
	1			57 (9.5)
	2			88 (14.7)
	3			219 (36.6)
	4			185 (30.9)
	≥5			49 (8.2)
**Living setting**				
	Large city			195 (32.6)
	Suburb			117 (19.6)
	Rural			107 (17.9)
	Large town			94 (15.7)
	Small town			84 (14)
	Other			1 (0.2)
**Average frequency of Facebook use**				
	Never			15 (2.5)
	Once a week			21 (3.5)
	2 to 3 times a week			52 (8.7)
	4 to 6 times a week			89 (14.9)
	Once a day			130 (21.7)
	Multiple times a day			289 (48.5)

### Awareness and Use of Facebook Well-being Features

**Figure 2:**
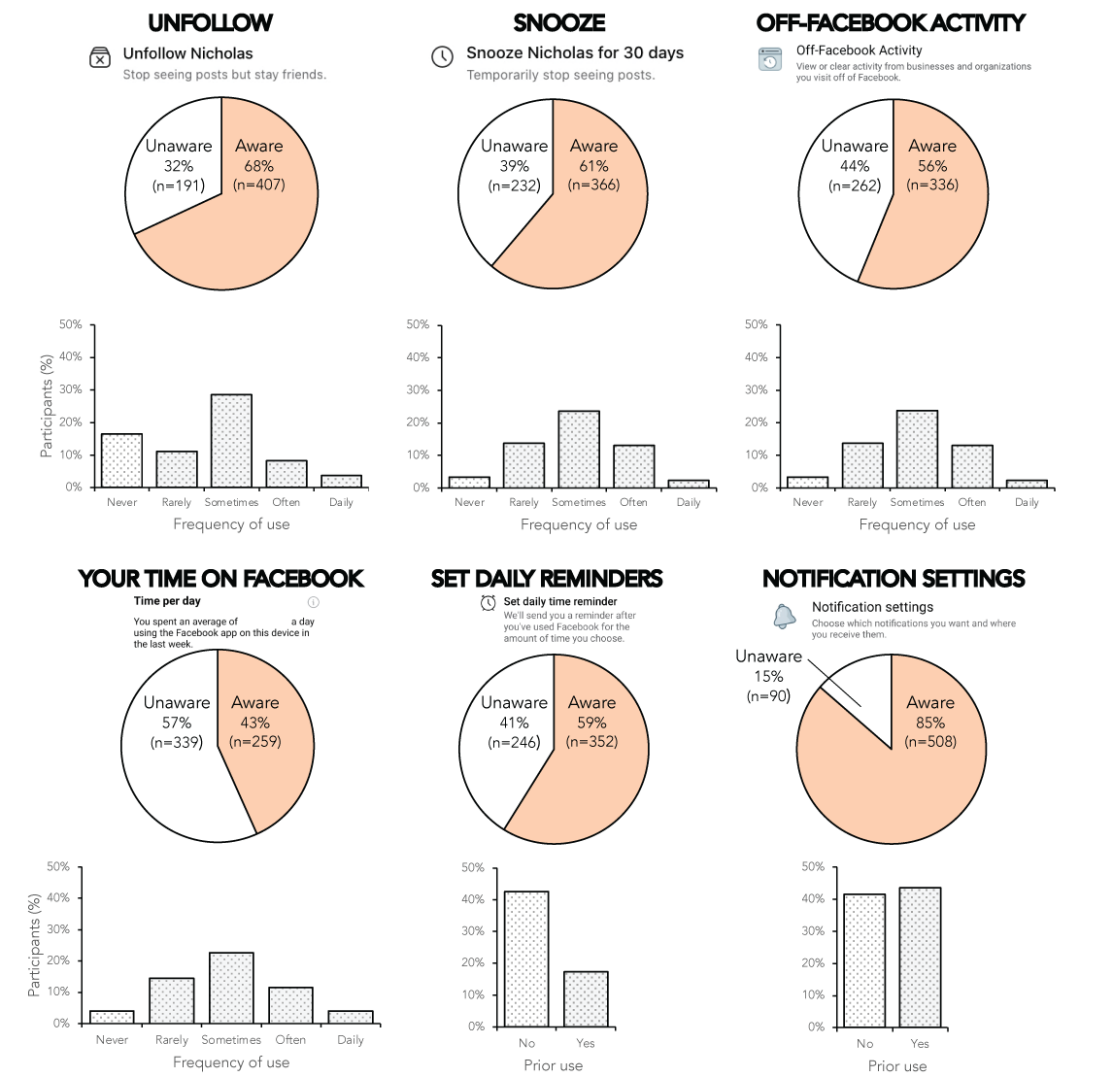
Participants indicated their awareness and usage of Facebook’s in-app digital well-being tools (*Unfollow, Snooze, Off-Facebook Activity, Your Time on Facebook, Set Daily Reminders,* and *Notification Settings*).

Of the 598 participants, 580 (97%, 95% CI 95.6%-98.4%) were aware of at least one of Facebook’s well-being features (Figure 2). However, awareness levels differed across features. For example, while 508 (85%, 95% CI 82.1%-87.9%) had heard of the *Notification Settings* feature, only 259 (43.3%, 95% CI 39.7%-47.3%) of participants knew about the *Your Time on Facebook* feature.

In terms of usage, of the 598 participants, 332 (55.5%, 95% CI 51.5%-59.5%) had used the *Snooze* feature, 316 (52.8%, 95% CI 48.7%-56.7%) had used the *Off-Facebook Activity Tracker* feature, 315 (52.7%, 95% CI 48.6%-56.6%) had used the *Your Time on Facebook* feature, and 309 (51.7%, 95% CI 47.7%-55.7%) had used the *Unfollow* feature. Less than half had adjusted *Notification Settings* (n=260, 43.5%, 95% CI 39.5%-47.5%), and fewer still had used the *Set Time Reminder* feature (n=104, 17.4%, 95% CI 14.4%-20.4%). Where the repeated use of features was possible, participants were most likely to report using them “sometimes” on an ad hoc basis rather than on a routine basis (based on the median ratings for *Snooze*, *Off-Facebook Activity*, *Your Time on Facebook*, and *Unfollow*; Figure 2).

### Use of Facebook Well-being Features and Psychological Symptoms

For the primary research question, we sought to predict participants’ depression, anxiety, and stress scores as a function of whether they used Facebook’s well-being features.

#### Depression

In terms of depression, we first replicated the well-documented association between Facebook usage and depression symptoms; namely, the more time that participants spent using Facebook, the higher their depression scores (model 1: β=2.754; *P*<.001; model 2: β=1.357; *P*<.001; model 3: β=1.586; *P*<.001; Table 2).

Factoring whether participants used Facebook’s well-being features increased the amount of variance in depression scores accounted for, from 8.7% (model 1) to 29.1% (model 2). Although the use of the *Notification Settings* feature (β=−1.579; *P*=.003) and the *Unfollow* button (β=−1.319; *P*=.02) was associated with lower depression scores, the use of the *Off-Facebook Activity* feature (β=4.905; *P*<.001) and the *Snooze* function (β=2.337; *P*<.001) was associated with higher depression scores. There was no significant association between depression scores and participants’ use of either the *Your Time on Facebook* feature or the *Set Time Reminder* feature (smallest *P*=.48).

Each of these findings was robust, and they persisted even when demographic variables were controlled for in model 3.

**Table 2:** Predicting depression symptoms as a function of Facebook usage patterns.

Variable		Models (dependent variable: depression subscale scores [DASS-21^a^])		
		Model 1 (*R*^2^=0.87), β estimate (95% CI)	Model 2 (*R*^2^=0.291), β estimate (95% CI)	Model 3 (*R*^2^=0.342), β estimate (95% CI)
Time spent on Facebook (hours per day)^b^		2.754^c^ (2.036 to 3.471)	1.357^c^ (0.667 to 2.047)	1.586^c^ (0.878 to 2.294)
Use of *Notification Settings* feature		N/A^d^	−1.579^e^ (−2.634 to −0.525)	−1.413^e^ (−2.459 to −0.367)
Use of *Unfollow* feature		N/A	−1.319^f^ (−2.444 to −0.195)	−1.252^f^ (−2.369 to −0.135)
Use of *Off-Facebook Activity* feature		N/A	4.905^c^ (3.604 to 6.206)	5.256^c^ (3.894 to 6.617)
Use of *Snooze* feature		N/A	2.337^c^ (1.060 to 3.613)	2.398^c^ (1.141 to 3.656)
Use of *Your Time on Facebook* feature		N/A	0.420 (−0.753 to 1.592)	0.384 (−0.784 to 1.553)
Use of *Set Daily Reminders* feature		N/A	0.115 (−1.323 to 1.554*)*	0.130 (−1.311 to 1.572)
Age group (base: <35 years)		N/A	N/A	−0.725 (−1.740 to 0.290)
Gender (base: women)		N/A	N/A	−0.645 (−1.660 to 0.370)
**Race (base: White)**				
	Black or African American	N/A	N/A	−1.704^f^ (−3.123 to −0.286)
	Other	N/A	N/A	0.18 (−2.023 to 2.059)
**Religion (base: no religion)**				
	Catholic	N/A	N/A	−3.113^e^ (−5.036 to −1.191)
	Protestant	N/A	N/A	−1.391 (−3.027 to 0.244)
	Other	N/A	N/A	−2.129 (−4.522 to 0.265)
**Marital status (base: single)**				
	Married	N/A	N/A	−1.090 (-2.551 to 0.370)
	Other	N/A	N/A	0.820 (−2.259 to 3.898)
Education level		N/A	N/A	0.506 (−0.279 to 1.291)
Employment status (base: full-time employment)		N/A	N/A	−0.701 (−2.330 to 0.929)
Income level		N/A	N/A	−0.748^c^ (−1.204 to −0.292)
Household size		N/A	N/A	0.306 (−0.207 to 0.819)
**Living setting (base: rural)**				
	Large city	N/A	N/A	−1.495^f^ (−2.925 to −0.064)
	Suburb	N/A	N/A	−1.289 (−2.902 to 0.323)
	Large town	N/A	N/A	−0.677 (−2.343 to 0.988)
	Small town	N/A	N/A	−1.351 (−3.095 to 0.394)

^a^DASS-21: 21-item Depression, Anxiety, and Stress Scale.

^b^Log-transformed.

^c^Significant at the *P*<.001 level.

^d^N/A: not applicable.

^e^Significant at the *P*<.01 level.

^f^Significant at the *P*<.05 level.

#### Anxiety

As with depression symptoms, the duration of Facebook use predicted increased anxiety scores (model 1: β=4.331; *P*<.001; model 2: β=2.270; *P*<.001; model 3: β=2.1846; *P*<.001; Table 3).

Again, the inclusion of variables that coded for participants’ use of Facebook’s well-being features increased the amount of variance accounted for, from 13.8% in model 1 to 40.3% in model 2. Namely, while the use of the *Notification Shortcut Bar* (β=−2.387; *P*<.001) and *Unfollow* functions (β=−1.603; *P*=.02) emerged as protective factors, the use of the *Off-Facebook Activity* (β=6.760, *P*<.001) and *Snooze* functions (β=3.134; *P*<.001) predicted higher anxiety. We found no evidence that anxiety scores were linked to the use of either the *Your Time on Facebook* feature or the *Set Time Reminder* feature (smallest *P*=.07). Each of these findings persisted when we controlled for demographic variables in model 3.

**Table 3:** Predicting anxiety symptoms as a function of Facebook usage patterns.

Variable			Models (dependent variable: anxiety subscale scores [DASS-21^a^])				
			Model 1 (*R*^2^=0.138), β estimate (95% CI)		Model 2 (*R*^2^=0.403), β estimate (95% CI)		Model 3 (*R*^2^=0.452), β estimate (95% CI)
Time spent on Facebook (hours per day)^b^			4.331^c^ (3.459 to 5.203)		2.270^c^ (1.479 to 3.062)		2.184^c^ (1.376 to 2.992)
Use of *Notification Settings* feature			N/A^d^		−2.387^c^ (−3.596 to −1.177)		−2.064^c^ (−3.257 to −0.871)
Use of *Unfollow* feature			N/A		−1.603^e^ (−2.893 to −0.314)		−1.593^e^ (−2.868 to −0.319)
Use of *Off-Facebook Activity* feature			N/A		6.760^c^ (5.268 to 8.252)		6.151^c^ (4.598 to 7.705)
Use of *Snooze* feature			N/A		3.134^c^ (1.670 to 4.598)		3.326^c^ (1.891 to 4.760)
Use of *Your Time on Facebook* feature			N/A		1.225 (−0.120 to 2.569)		0.884 (−0.449 to 2.217)
Use of *Set Daily Reminders* feature			N/A		0.327 (−1.323 to 1.977)		0.078 (−1.556 to 1.723)
Age group (base: <35 years)			N/A		N/A		−0.996 (−2.155 to 0.162)
Gender (base: women)			N/A		N/A		−1.165^e^ (−2.323 to −0.006)
**Race (base: White)**							
	Black or African American	N/A		N/A		−1.227 (−2.846 to 0.391)	
	Other	N/A		N/A		−0.503 (−2.832 to 1.826)	
**Religion (base: no religion)**							
	Catholic	N/A		N/A		−1.864 (−4.057 to 0.329)	
	Protestant	N/A		N/A		0.027 (−1.839 to 1.894)	
	Other	N/A		N/A		−0.968 (−3.699 to 1.763)	
**Marital status (base: single)**							
	Other	N/A		N/A		−3.877^e^ (−7.390 to −0.365)	
	Married	N/A		N/A		−0.742 (−2.409 to 0.924)	
Education level			N/A		N/A		0.879 (−0.016 to 1.774)
Employment status (base: full-time employment)			N/A		N/A		−1.606 (−3.465 to 0.254)
Income level			N/A		N/A		−0.737^f^ (−1.258 to −0.217)
Household size			N/A		N/A		0.411 (−0.175 to 0.996)
**Living setting (base: rural)**							
	Large city	N/A		N/A		−1.958^e^ (−3.590 to −0.326)	
	Suburb	N/A		N/A		−2.398^e^ (−4.238 to −0.558)	
	Large town	N/A		N/A		−0.997 (−2.897 to 0.903)	
	Small town	N/A		N/A		−1.385 (−3.375 to 0.605)	

^a^DASS-21: 21-item Depression, Anxiety, and Stress Scale.

^b^Log-transformed.

^c^Significant at the *P*<.001 level.

^d^N/A: not applicable.

^e^Significant at the *P*<.05 level.

^f^Significant at the *P*<.01 level.

#### Stress

The time spent on Facebook was again linked to increased stress scores (model 1: β=3.851; *P*<.001; model 2: β=1.825; *P*<.001; model 3: β=2.103; *P*<.001; Table 4).

**Table 4:** Predicting stress symptoms as a function of Facebook usage patterns.

Variable			Models (dependent variable: stress subscale scores [DASS-21^a^])				
			Model 1 (*R*^2^=0.083), β estimate (95% CI)		Model 2 (*R*^2^=0.299), β estimate (95% CI)		Model 3 (*R*^2^=0.353), β estimate (95% CI)
Time spent on Facebook (hours per day)^b^			3.851^c^ (2.822 to 4.881)		1.825^c^ (0.843 to 2.806)		2.103^c^ (1.098 to 3.108)
Use of *Notification Settings* feature			N/A^d^		−2.980^c^ (−4.480 to −1.480)		−2.677^c^ (−4.161 to −1.193)
Use of *Unfollow* feature			N/A		−1.731^e^ (−3.331 to −0.132)		−1.778^e^ (−3.363 to −0.193)
Use of *Off-Facebook Activity* feature			N/A		7.124^c^ (5.274 to 8.974)		7.321^c^ (5.388 to 9.253)
Use of *Snooze* feature			N/A		2.845^f^ (1.030 to 4.660)		3.015^c^ (1.231 to 4.800)
Use of *Your Time on Facebook* feature			N/A		1.297 (−0.371 to 2.964)		1.251 (−0.407 to 2.909)
Use of *Set Daily Reminders* feature			N/A		−0.453 (−2.499 to 1.593)		−0.314 (−2.360 to 1.732)
Age group (base: <35 years)			N/A		N/A		−1.362 (−2.803 to 0.079)
Gender (base: women)			N/A		N/A		−1.321 (−2.761 to 0.120)
**Race (base: White)**							
	Black or African American	N/A		N/A		−2.051^e^ (−4.064 to −0.038)	
	Other	N/A		N/A		1.260 (−1.637 to 4.156)	
**Religion (base: no religion)**							
	Catholic	N/A		N/A		−4.459^c^ (−7.187 to −1.731)	
	Protestant	N/A		N/A		−1.621 (−3.942 to 0.700)	
	Other	N/A		N/A		−1.575 (−4.972 to 1.822)	
**Marital status (base: single)**							
	Married	N/A		N/A		−1.336 (−3.408 to 0.737)	
	Other	N/A		N/A		−2.086 (−6.455 to 2.283)	
Education level			N/A		N/A		0.971 (−0.143 to 2.084)
Employment status (base: full-time employment)			N/A		N/A		−1.090 (−3.403 to 1.223)
Income level			N/A		N/A		−0.897^f^ (−1.544 to −0.250)
Household size			N/A		N/A		0.241 (−0.488 to 0.969)
**Living setting (base: rural)**							
	Large city	N/A		N/A		−2.148^e^ (−4.178 to −0.118)	
	Suburb	N/A		N/A		−2.251 (−4.540 to 0.037)	
	Large town	N/A		N/A		−0.674 (−3.037 to 1.689)	
	Small town	N/A		N/A		−1.324 (−3.799 to 1.152)	

^a^DASS-21: 21-item Depression, Anxiety, and Stress Scale.

^b^Log-transformed.

^c^Significant at the *P*<.001 level.

^d^N/A: not applicable.

^e^Significant at the *P*<.05 level.

^f^Significant at the *P*<.01 level.

When we added participants’ use of Facebook features as predictors, the amount of variance accounted for increased from 8.3% (model 1) to 29.9% (model 2). Once again, we found that participants who used the *Notification Shortcut Bar* function (β=−2.980; *P*<.001) and *Unfollow* button (β=−1.731; *P*=.03) had lower stress scores, but those who used the *Off-Facebook Activity* feature (β=7.124; *P*<.001) and the *Snooze* button (β=2.845; *P*=.002) had higher stress scores. These associations remained significant in model 3, for which demographic variables were controlled. Across both models 2 and 3, there were no significant associations between stress scores and the use of either the *Your Time on Facebook* feature or the *Set Time Reminder* feature (smallest *P*=.13).

#### Sensitivity Analyses

For our sensitivity analyses, we (1) repeated models 2 and 3 without factoring participants’ duration of Facebook use and (2) reran model 3, with *age group* entered as an ordinal variable (using the following age categories: 21-25, 26-35, 36-45, 46-55, ≥56 years). As shown in Tables S1-S3 in Multimedia Appendix 1 and Tables S1-S3 in Multimedia Appendix 2, the key findings pertaining to Facebook’s digital well-being tools did not change.

## Discussion

### Principal Findings

In this paper, we present the first empirical study to evaluate Facebook’s digital well-being tools. First, echoing prior studies [8], we found that participants who spent more time on Facebook had more symptoms of depression, anxiety, and stress. Accounting for Facebook consumption alone explained one-tenth of the variance (range 8%-13%) in participants’ well-being. Consequently, we examined (1) whether participants used the platform’s digital well-being tools and (2) whether usage was associated with better mental health.

Although most participants (580/598, 97%) knew about Facebook’s well-being tools, each tool was used by only 17.4% (104/598) to 55.5% (332/598) of participants largely on an ad hoc basis. These adoption rates are lower than those of mainstream Facebook features that were introduced much earlier to the platform. For example, an estimated 4 in 5 Facebook users have deployed the *Unfriend* feature to remove contacts [27], while 3 in 4 have used the *Untag* feature to remove their name from a photograph [28].

Participants who used either the *Notification Settings* feature or the *Unfollow* tools reported fewer symptoms of depression, anxiety, and stress. Conversely, those who used either the *Snooze* feature or the *Off-Facebook Activity* feature had higher scores on each of these measures. Finally, there was no evidence that the *Your Time on Facebook* feature or the *Set Daily Reminders* feature was associated with well-being. This set of findings was robust and was observed regardless of whether we controlled for participants’ duration of Facebook use or their sociodemographic factors.

Taken together, our findings underscore the complexity of designing social media platforms to optimize user welfare. Of the 6 digital well-being tools we examined, only 2 were associated with a decreased risk for mental health symptoms—(1) a feature for toning down the amount of content that is brought to a user’s attention (*Notification Shortcut Bar* function) and (2) a feature that allows users to customize their news feeds (*Unfollow* feature), which, in theory, minimizes the amount of social comparisons made on the platform. Nonetheless, it remains unclear why two other features that supported the customization of news feeds (*Snooze* and *Off-Facebook Activity*) predicted a higher risk for mental health symptoms. Further research is thus needed to understand these patterns.

It is noteworthy that we found no significant associations between the use of time-monitoring features (*Your Time on Facebook* and *Set Daily Reminders*) and well-being. This finding is counterintuitive because the time spent on Facebook has been linked repeatedly to poorer mental health outcomes (including in this study) [8]. Consequently, most social media developers have incorporated time-monitoring features into their digital well-being programs, allowing users to track how much time they have spent on a platform or set limits on usage (eg, on YouTube, Instagram, Facebook, and TikTok) [13,16,29]. Nonetheless, we found no empirical support for this widely used strategy, consistent with “digital detox” studies reporting that interventions for curbing social media use have a limited impact on mood and well-being [30].

### Implications

Moving forward, our study has several implications for research and practice. First, it appears that the current well-being measures taken by social media platforms may be insufficient. This begs the question of how digital well-being tools should be designed to maximize users’ benefits. Despite widespread calls for app developers to prioritize their users, there remains limited empirical data for guiding platforms in carrying out this mandate. We thus urge researchers to address this gap, thereby allowing for an evidence-based toolkit of in-app well-being features to be developed.

### Limitations

In reporting our findings, we noted several limitations of our study. First, we chose the design of an epidemiological survey [31,32]. In a new area of research, this allowed us to (1) document baseline adoption rates for digital well-being tools and (2) examine multiple tools at the same time. Nonetheless, correlation does not equate to causation, and our findings need to be followed up with randomized controlled trials. Second, we recruited participants within the general population of internet users; the participant demographics were comparable to that of US Facebook users [33]. Nonetheless, it is possible that stronger effects would be observed in vulnerable groups, such as among individuals with problematic forms of Facebook usage [34] or among adolescents. Further research should thus explore this possibility. Finally, we focused on Facebook because of its widespread popularity. It is currently unclear whether our findings would generalize to other social networking services (eg, Instagram).

### Conclusion

In the 2022 State of the Union Address, President Joe Biden called for social media platforms to be held accountable and for companies to pursue users’ benefits over profits [35]. Amid these petitions, there is a need to understand how social media platforms can be designed to optimize users’ well-being. Accordingly, our study provides the first line of evidence that two digital well-being features may be linked to improved mental health. At the same time, we also caution app developers that (1) not all well-being features are alike and (2) certain features could backfire. Moving forward, we urge further research to develop and carefully investigate the impact of digital well-being tools on social media.

## Appendix

Multimedia Appendix 1 Sensitivity analyses.

Multimedia Appendix 2 Sensitivity analyses: regression analyses with age group as an ordinal variable.

## Abbreviations

DASS-21: 21-item Depression, Anxiety, and Stress Scale

## References

[1] Exposure Labs (2020). The social dilemma. Netflix.

[2] Wells G, Horwitz J, Seetharaman D (2021). The Facebook files: Facebook knows Instagram is toxic for teen girls, company documents show. The Wall Street Journal.

[3] Ivie EJ, Pettitt A, Moses LJ, Allen NB (2020). A meta-analysis of the association between adolescent social media use and depressive symptoms. J Affect Disord.

[4] Cunningham S, Hudson CC, Harkness K (2021). Social media and depression symptoms: a meta-analysis. Res Child Adolesc Psychopathol.

[5] Huang C (2022). A meta-analysis of the problematic social media use and mental health. Int J Soc Psychiatry.

[6] Huang C (2017). Time spent on social network sites and psychological well-being: A meta-analysis. Cyberpsychol Behav Soc Netw.

[7] Marino C, Gini G, Vieno A, Spada MM (2018). A comprehensive meta-analysis on problematic Facebook use. Comput Human Behav.

[8] Yoon S, Kleinman M, Mertz J, Brannick M (2019). Is social network site usage related to depression? A meta-analysis of Facebook-depression relations. J Affect Disord.

[9] Yang FR, Wei CF, Tang JH (2019). Effect of Facebook social comparison on well-being: A meta-analysis. Journal of Internet Technology.

[10] Bhargava VR, Velasquez M (2020). Ethics of the attention economy: The problem of social media addiction. Bus Ethics Q.

[11] Giraldo-Luque S, Afanador PNA, Fernández-Rovira C (2020). The struggle for human attention: Between the abuse of social media and digital wellbeing. Healthcare (Basel).

[12] Nix N (2021). Instagram to nudge people to ‘Take a Break’ from scrolling. Bloomberg.

[13] How does YouTube support users' digital wellbeing?. YouTube.

[14] Number of monthly active Facebook users worldwide as of 2nd quarter 2022 (in millions). Statista.

[15] Song H, Zmyslinski-Seelig A, Kim J, Drent A, Victor A, Omori K, Allen M (2014). Does Facebook make you lonely?: A meta analysis. Comput Human Behav.

[16] Ranadive A, Ginsberg D (2018). New tools to manage your time on Facebook and Instagram. Meta.

[17] Facebook help center. Facebook.

[18] Baysha O (2020). Dividing social networks: Facebook unfriending, unfollowing, and blocking in turbulent political times. Russian Journal of Communication.

[19] Wu TY (2021). Proactive opinion expression avoidance about same-sex marriage on social media: Acceptance, reactance, and self-censorship. Mass Commun Soc.

[20] Wu TY, Xu X, Atkin D (2020). The alternatives to being silent: exploring opinion expression avoidance strategies for discussing politics on Facebook. Internet Research.

[21] Gashi L, Knautz K (2016). Unfriending, hiding and blocking on Facebook.

[22] Gashi L, Knautz K (2015). ‘Somebody that I used to know’ – Unfriending and becoming unfriended on Facebook.

[23] Predicting psychological symptoms when Facebook’s digital well-being features are used: A cross-sectional survey. Center for Open Science.

[24] Nisar TM, Prabhakar G, Ilavarasan PV, Baabdullah AM (2019). Facebook usage and mental health: An empirical study of role of non-directional social comparisons in the UK. Int J Inf Manage.

[25] Lovibond SH, Lovibond PF (1995). Manual for the Depression Anxiety Stress Scales, 2nd Edition.

[26] Hauser D, Paolacci G, Chandler J, Kardes FR, Herr PM, Schwarz N (2019). Common concerns with MTurk as a participant pool: Evidence and solutions. Handbook of Research Methods in Consumer Psychology.

[27] Verswijvel K, Heirman W, Hardies K, Walrave M (2018). Adolescents' reasons to unfriend on Facebook. Cyberpsychol Behav Soc Netw.

[28] Lang C, Barton H (2015). Just untag it: Exploring the management of undesirable Facebook photos. Comput Human Behav.

[29] Digital well-being. TikTok.

[30] Radtke T, Apel T, Schenkel K, Keller J, von Lindern E (2021). Digital detox: An effective solution in the smartphone era? A systematic literature review. Mob Media Commun.

[31] Saw YE, Tan EYQ, Liu JS, Liu JC (2021). Predicting public uptake of digital contact tracing during the COVID-19 pandemic: Results from a nationwide survey in Singapore. J Med Internet Res.

[32] Liu JCJ, Tong EMW (2020). The relation between official WhatsApp-distributed COVID-19 news exposure and psychological symptoms: Cross-sectional survey study. J Med Internet Res.

[33] Distribution of Facebook users worldwide as of January 2022, by age and gender. Statista.

[34] Andreassen CS, Torsheim T, Brunborg GS, Pallesen S (2012). Development of a Facebook addiction scale. Psychol Rep.

[35] (2022). State of the Union Address. The White House.

