# Supplementary material for: Predicting Psychological Symptoms When Facebook’s Digital Well-being Features Are Used: Cross-sectional Survey Study
Source: JMIR Form Res. 2022 Aug 29;6(8):e39387. doi: 10.2196/39387 (PMC9468917; doi:10.2196/39387)
Supplement: Multimedia Appendix 2 [file formative_v6i8e39387_app2.docx]

**Appendix 2**

In Tables S1-S3, we repeated the regression analyses with age group coded as an ordinal variable (with 5 age categories instead of 2). Our primary conclusions did not change.

*Table S1.* Predicting depression symptoms as a function of Facebook usage patterns.

| Dependent variable: Depression symptoms (DASS-21)^a^ | |  |
| --- | --- | --- |
|  | (A)^b^ | |
| Time spent on Facebook (hours per day)^c^ | 1.614***  (0.910, 2.317) | |
| Use of ‘Notification Settings’ | -1.390**  (-2.429, -0.352) | |
| Use of ‘Unfollow’ | -1.251*  (-2.360, -0.141) | |
| Use of ‘Off-Facebook Activity’ | 5.234***  (3.880, 6.587) | |
| Use of ‘Snooze’ | 2.359***  (1.110, 3.607) | |
| Use of ‘Your Time on Facebook’ | 0.350  (-0.811, 1.512) | |
| Use of ‘Set Daily Reminders’ | 0.075  (-1.358, 1.509) | |
| Age group | -0.686  (-1.139, -0.232) | |
| Gender *(base = female)* | -0.559  (-1.569, 0.451) | |
| Race *(base = white)* |  | |
| Black or African American | -1.877**  (-3.293, -0.462) | |
| Others | -0.051  (-2.080, 1.979) | |
| Religion *(base = no religion)* |  | |
| Catholic | -3.164**  (-5.074, -1.253) | |
| Protestant | -1.335  (-2.962, 0.291) | |
| Others | -2.009  (-4.389, 0.371) | |
| Marital status *(base = single)* |  | |
| Married | -0.816  (-2.271, 0.639) | |
| Others | 1.312  (-1.739, 4.362) | |
| Education level | 0.550  (-0.230, 1.331) | |
| Employment status (*base = full-time employment)* | -0.599  (-2.218, 1.021) | |
| Income level | -0.769***  (-1.222, -0.315) | |
| Household size | 0.268  (-0.243, 0.779) | |
| Living setting *(base = rural)* |  | |
| Large city | -1.493*  (-2.915, -0.071) | |
| Suburb | -1.300  (-2.903, 0.303) | |
| Large town | -0.752  (-2.408, 0.904) | |
| Small town | -1.345  (-3.079, 0.388) | |
| *R^2^* | .350 | |

^a^Dependent variable: Depression subscale scores from the 21-item Depression Anxiety Stress Scale (DASS-21). Data reported as beta estimates (95% CI).

^b^Corresponds to Model 3 of Table 3.

^c^Log-transformed

****P* < .001, ***P* < .01, **P* < .05

*Table S2.* Predicting anxiety symptoms as a function of Facebook usage patterns.

| Dependent variable: Anxiety symptoms (DASS-21)^a^ | |  |
| --- | --- | --- |
|  | (A)^b^ | |
| Time spent on Facebook (hours per day)^c^ | 2.219***  (1.415, 3.022) | |
| Use of ‘Notification Settings’ | -2.049***  (-3.236, -0.862) | |
| Use of ‘Unfollow’ | -1.585*  (-2.852, -0.317) | |
| Use of ‘Off-Facebook Activity’ | 6.130***  (4.584, 7.676) | |
| Use of ‘Snooze’ | 3.271***  (1.845, 4.697) | |
| Use of ‘Your Time on Facebook’ | 0.844  (-0.483, 2.170) | |
| Use of ‘Set Daily Reminders’ | 0.014  (-1.623,1.651) | |
| Age group | -0.765*  (-1.283, -0.247) | |
| Gender *(base = female)* | -1.086*  (-2.240, -0.067) | |
| Race (*base = white)* |  | |
| Black or African American | -1.416  (-3.033, 0.201) | |
| Others | -0.584  (-2.902, 1.734) | |
| Religion *(base = no religion)* |  | |
| Catholic | -1.904  (-4.086, 0.278) | |
| Protestant | 0.082  (-1.775, 1.940) | |
| Others | -0.858  (-3.577, 1.860) | |
| Marital status *(base = single)* |  | |
| Others | -3.459  (-6.943, 0.025) | |
| Married | -0.496  (-2.158, 1.166) | |
| Education level | 0.926*  (0.034, 1.817) | |
| Employment status *(base = full-time employment)* | -1.518  (-3.368, 0.332) | |
| Income level | -0.757**  (-1.275, -0.239) | |
| Household size | 0.370  (-0.214, 0.953) | |
| Living setting (*base = rural)* |  | |
| Large city | -1.951*  (-3.575, -0.327) | |
| Suburb | -2.413**  (-4.244, -0.582) | |
| Large town | -1.067  (-2.959, 0.824) | |
| Small town | -1.372  (-3.352, 0.608) | |
| *R^2^* | 0.457 | |

^a^Dependent variable: Anxiety subscale scores from the 21-item Depression Anxiety Stress Scale (DASS-21). Data reported as beta estimates (95% CI).

^b^Corresponds to Model 3 of Table 4.

^c^Log-transformed

***P < .001, **P < .01, *P < .05

*Table S3.* Predicting stress symptoms as a function of Facebook usage patterns.

| Dependent variable: Stress symptoms (DASS-21)^a^ | |
| --- | --- |
|  | (A)^b^ |
| Time spent on Facebook (hours per day)^c^ | 2.153***  (1.156, 3.149) |
| Use of ‘Notification Settings’ | -2.646***  (-4.117, -1.174) |
| Use of ‘Unfollow’ | -1.770*  (-3.342, -0.198) |
| Use of ‘Off-Facebook Activity’ | 7.286***  (5.369, 9.203) |
| Use of ‘Snooze’ | 2.940***  (1.172, 4.708) |
| Use of ‘Your Time on Facebook’ | 1.191  (-0.454, 2.836) |
| Use of ‘Set Daily Reminders’ | -.409  (-2.439, 1.620) |
| Age group | -1.165***  (-1.807, -.0522) |
| Gender *(base = female)* | -1.187  (-2.617, 0.243) |
| Race *(base = white)* |  |
| Black or African American | -2.341*  (-4.346, -0.337) |
| Others | 1.141  (-1.733, 4.014) |
| Religion *(base = no religion)* |  |
|  |  |
| Catholic | -4.533***  (-7.239, -1.828) |
| Protestant | -1.532  (-3.835, 0.772) |
| Others | -1.388  (-4.758, 1.983) |
| Marital status *(base = single)* |  |
|  |  |
| Married | -0.911  (-2.972, 1.150) |
| Other | -1.341  (-5.661, 2.980) |
| Education level | 1.044  (-0.062, 2.150) |
| Employment status (*base = full-time employment)* | -0.935  (-3.229, 1.358) |
| Income level | -0.930**  (-1.572, -0.287) |
| Household size | 0.177  (-0.546, 0.901) |
| Living setting *(base = rural)* |  |
| Large city | -2.142*  (-4.155, -0.128) |
| Suburb | -2.271*  (-4.542, -0.001) |
| Large town | -0.792  (-3.137, 1.554) |
| Small town | -1.310  (-3.765, 1.145) |
| *R^2^* | .364 |

^a^Dependent variable: Stress subscale scores from the 21-item Depression Anxiety Stress Scale (DASS-21). Data reported as unstandardized estimates (95% CI).

^b^Corresponds to Model 3 of Table 5.

^c^Log-transformed

****P* < .001, ***P* < .01, **P* < .05
